# Simultaneous monitoring of the middle cerebral and basilar arteries to detect right-to-left shunts using transcranial Doppler by agitated saline administration

**DOI:** 10.1038/s41598-022-10645-7

**Published:** 2022-04-22

**Authors:** Min Kim, So Young Park, Ji Man Hong

**Affiliations:** Department of Neurology, School of Medicine, Ajou University, 164, World cup-ro, Yeongtong-gu, Suwon-si, Gyeonggi-do 16499 Republic of Korea

**Keywords:** Medical research, Neurology

## Abstract

Transcranial Doppler (TCD) is an easy, non-invasive, and real-time monitoring device for detecting right-to-left shunts (RLS). Nonetheless, it has limited benefits in patients with poor temporal windows. Therefore, we aimed to investigate whether the basilar artery (BA) window was as effective as the middle cerebral artery (MCA) in detecting RLS during TCD monitoring. Overall, we enrolled 344 patients with stroke, transient ischemic attack, headache, or dizziness. MCA and BA were monitored using a modified headset. To investigate the feasibility of the suboccipital window in detecting RLS, we instituted an evaluation tool with three tiers to evaluate microembolic signals (MESs) during TCD monitoring. Tier 1: TCD monitoring of the MCA (bilaterally) in the resting state, tier 2: TCD monitoring of the MCA (bilaterally) while performing the Valsalva maneuver, and tier 3: TCD monitoring of the index MCA and BA while performing the Valsalva maneuver. In tiers 2 and 3, a high agreement rate of 0.808 and 0.809 (p < 0.001), respectively, on the weighted kappa index, and a high intra-class correlation coefficient of 0.982 and 0.986 (p < 0.001), respectively, were observed on detecting MESs. Our data suggests that the BA window is as effective as the MCA window for detecting RLS on TCD.

## Introduction

Ischemic stroke without risk factors for cerebral vascular disease should be evaluated for alternative causes^1^. The causes of 10–40% of all strokes are not identified despite comprehensive diagnostic testing^2^. These strokes are known as cryptogenic strokes. The most common cause of a cryptogenic stroke is probably a paradoxical embolism due to a patent foramen ovale (PFO) or an atrial septal aneurysm (ASA)^3^. Right-to-left shunts (RLS) through PFOs or ASAs cause paradoxical embolisms, which originate in the systemic venous circulation and enter the systemic arterial circulation. Recent studies have shown that PFO closure reduces the recurrence rate of cryptogenic stroke^4–7^. Therefore, it is essential to detect the presence of RLS through PFOs in cryptogenic stroke.

Transesophageal echocardiography (TEE) is a gold standard modality for detecting cardiac RLS^8–10^. However, the use of TEE may be limited in patients with acute stroke who have acute illness, mental status deteriorates, and coagulopathy/bleeding tendency^11–13^. Transcranial Doppler (TCD) is an easy, non-invasive, and real-time monitoring apparatus for detecting RLS^14,15^. Additionally, this technique is highly sensitive, specific, and convenient for screening compared to TEE regardless of location^16,17^. However, a limitation of TCD is that some patients cannot be evaluated because of a poor temporal window (PTW)^18^. In a previous study, 5–37% of patients had inadequate TCD because of a PTW^19–23^. Also numerous studies showed that PTW is more common in older women and non-Caucasians^24–27^. Especially, Patients with a PTW account for 29–34% of the Asian population, which is a much higher incidence than that of the Caucasian population^19,20,28^. Interestingly, in patients with provoked RLS, the ischemic lesion was found to be located predominantly in the cerebral posterior circulation system^29^. Several studies have attempted to utilize the suboccipital window for detecting RLS on TCD in patients with a PTW^30–32^. These studies showed that usefulness of suboccipital window using vertebral artery (VA) to replace temporal window. However, the usefulness of the suboccipital window has not been elucidated by quantitative detection of agitated saline signals especially in basilar artery (BA). Therefore, the purpose of this study was to investigate if simultaneous monitoring of the BA was as effective as monitoring the MCA for detecting RLS on TCD.

## Results

### Demographic characteristics

The mean age of the patients was 58 ± 16 years, and the male: female ratio was 165:179. Among the patients, 129 (37.5%) had hypertension, 70 (20.3%) had diabetes mellitus, and 92 (26.7%) had dyslipidemia. The reasons for the test were stroke (n = 229), headache (n = 53), transient ischemic attack (TIA) (n = 22), white matter hyperintensities (WMHI) (n = 20), transient global amnesia (TGA) (n = 11), and dizziness (n = 9). Among these patients, 53 (15.4%) had PTWs; 41 had PTWs on both sides and 12 had it on one side only (eight on the right side and four on the left side) detail the demographic characteristics of the patients in this study are shown in Table 1.

**Table 1:** Demographics of enrolled patients.

	Patients (n = 344)		
**Demographic characteristics**			
Age (years)	58 ± 16 (13–89)		
Sex (male/female)	165/179		
Poor temporal window, n (%)	53 (15.4)^a^		
**Underlying disease**			
Hypertension, n (%)	129 (37.5)		
Diabetes mellitus, n (%)	70 (20.3)		
Dyslipidemia, n (%)	92 (26.7)		
History of stroke, n (%)	242 (70.3)		
Cardiac problem, n (%)	7 (2.0)		
**Reasons for testing using the TCD**			
Stroke, n (%)	229 (66.6)		
Headache, n (%)	53 (15.4)		
TIA, n (%)	22 (6.4)		
WMHI, n (%)	20 (5.8)		
TGA, n (%)	11 (3.2)		
Dizziness, n (%)	9 (2.6)		

Baseline laboratory findings	Stroke (n = 229)	Non-stroke (n = 115)	p-value
Hemoglobin	13.9 ± 7.0	14.0 ± 1.31	0.905
Platelet	239.2 ± 75.0	231.66 ± 48.7	0.287
HbA1c	6.0 ± 1.1	5.8 ± 1.3	0.214
Total cholesterol	173.0 ± 149.8	181.5 ± 40.9	0.579
CRP	0.6 ± 1.7	0.6 ± 2.1	0.844

*TIA* transient ischemic attack, *WMHI* white matter hyperintensities, *TGA* transient global amnesia, *HbA1c* hemoglobin A1c, *CRP* C-reactive protein.

^a^Poor temporal window = Bilateral 41; Right 8; left 4.

### Outcomes of the TCD monitoring parameters in both of the MCAs while performing the Valsalva maneuver in Tier 2

**Figure 1:**
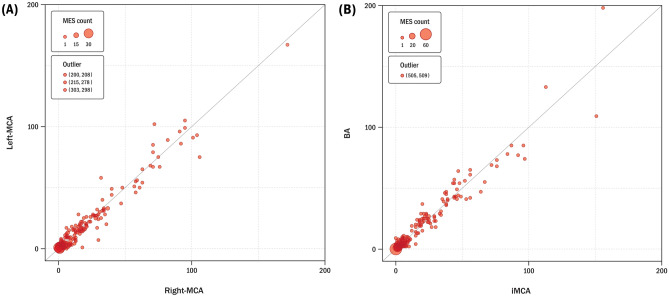
Comparison of TCD-PFO in the MCAs bilaterally or in the MCA and BA. (**A**) The bubble counts in the MCAs bilaterally on TCD (Tier 2), ICC = 0.982 (p < 0.001). (**B**) The bubble counts in the iMCA and BA on TCD (Tier 3), ICC = 0.986 (p < 0.001).

A total of 344 patients were measured simultaneously in both the MCAs of TCD monitoring with Valsalva maneuver in Tier2. In 41 patients with bilateral PTWs, bilateral internal carotid artery (ICA) was used instead of MCA on TCD examination. Also, in 12 patients with unilateral PTW, unilateral ICA and contralateral MCA were used on TCD monitoring (Table 1). When measured simultaneously in both of the MCAs, the average number of microembolic signals (MESs) were 14.97 ± 31.18 (range 0–298) in the right MCA, and 14.94 ± 32.41 (range 0–303) in the left MCA in tier 2. The measured MESs was categorized using the International Consensus Criteria or Spencer’s Logarithmic Scale (SLS) in both the MCAs, and we compared the concordance of right and left MCA using weight kappa index. The weighed kappa index was 0.687 (p < 0.001) in both the MCAs when assessed using the International Consensus Criteria (Table 2). In addition, the weighed kappa index was 0.808 (p < 0.001) when both the MCAs were evaluated using the SLS (Table 3). Moreover, when we had compared the degree of agreement on count of MESs in both the MCA using the intraclass correlation coefficient (ICC), the ICC was 0.982 (p < 0.001) on comparing the number of MESs measured in both MCAs (Fig. 1).

**Table 2:** Categorization as per the International Consensus Criteria in tiers 2 and 3.

Tier 2 (R-MCA vs L-MCA)	Left (L)				Total
	Grade 0	Grade 1	Grade 2	Grade 3	
**Right (R)**					
Grade 0	22	48	0	0	70
Grade 1	46	106	8	0	160
Grade 2	0	13	98	0	111
Grade 3	0	0	0	3	3
Total	68	167	106	3	344
		Weighted Kappa index 0.687 (p < 0.001)			

Tier 3 (iMCA vs BA)	BA				Total
	Grade 0	Grade 1	Grade 2	Grade 3	
**iMCA**					
Grade 0	78	18	0	0	96
Grade 1	17	104	1	0	122
Grade 2	0	2	78	0	80
Grade 3	0	0	0	5	5
Total	95	124	79	5	303
		Weighted Kappa index 0.812 (p < 0.001)			

*L-MCA* left middle cerebral artery, *R-MCA* right middle cerebral artery, *iMCA* index of middle cerebral artery, *BA* basilar artery.

**Table 3:** Categorization as per the Spencer logarithmic scale in tiers 2 and 3.

Tier 2 (R-MCA vs L-MCA)	Left (L)						Total
	0	I	II	III	IV	V	
**Right (R)**							
0	22	48	0	0	0	0	70
I	46	106	8	0	0	0	160
II	0	13	53	2	0	0	68
III	0	0	4	32	2	0	38
IV	0	0	0	3	4	0	7
o	0	0	0	0	1	0	1
Total	68	167	65	37	7	0	344
		Weighted Kappa index 0.808 (p < 0.001)					

Tier 3 (iMCA vs BA)	BA						Total
	0	I	II	III	IV	V	
**iMCA**							
0	78	18	0	0	0	0	96
I	17	104	1	0	0	0	122
II	0	2	39	1	0	0	42
III	0	0	2	37	0	0	39
IV	0	0	0	0	3	0	3
V	0	0	0	0	0	1	1
Total	95	124	42	38	3	1	303
		Weighted Kappa index 0.809 (p < 0.001)					

*L-MCA* left middle cerebral artery, *R-MCA* right middle cerebral artery, *iMCA* index of middle cerebral artery, *BA* basilar artery.

### Outcomes of the TCD monitoring parameters in both the index MCA and BA while performing the Valsalva maneuver in Tier 3

The index MCA(iMCA) and BA were measured in 303 patients, after excluding 41 out of 344 patients due to PTWs on both sides. Out of a total of 303 patients, 182 (60.1%) on the right and 121 (39.9%) on the left were measured simultaneously with the BA. When MESs was measured simultaneously, the average number of bubbles was 14.15 ± 36.32 (range 0–505) in the iMCA and 14.35 ± 38.85 (range 0–559) in the BA.

When iMCA and BA were categorized using the International Consensus Criteria to evaluate the concordance, The weighted kappa index was 0.812 (p < 0.001) when estimated (Table 2). Also, the weighted kappa index was 0.807 (p < 0.001) when iMCA and BA were evaluated using the SLS (Table 3). Also, when we had compared the degree of agreement on count of MESs in the iMCA and BA using the ICC, the ICC was 0.986 (p < 0.001) in the iMCA and BA (Fig. 1). The measurements for both the MCA and BA were similar when compared with the MCA measurements bilaterally.

## Discussion

This study shows that the suboccipital window for insonation of the BA is as effective as the temporal window for MCA in detecting RLS using the TCD.

Approximately 5–37% of stroke patients have PTWs that prevent sufficient TCD insonation of cerebral vessels around the circle of Willis^19–23^. The PTW rates are higher in Asians than in Caucasians^17,19^. It was difficult to evaluate RLS using TCD in patients with PTWs. Therefore, some studies had attempted to detect RLS using the suboccipital windows, submandibular ICA, or the orbital windows rather than the temporal windows^30–33^. The advantages of using agitated saline while performing the TCD in a suboccipital window (for insonation of the BA) instead of the submandibular window or the orbital windows can be explained by several reasons in our study.

First of all, this study is meaningful in that the suboccipital window using BA has the similar diagnostic value as the temporal window using MCA to quantitatively detect RLS. One study identified MESs using the orbital window for ICA and the temporal window for MCA to detect RLS, and not the suboccipital window in a TCD study^33^. Another study compared the monitoring of cervical arteries (submandibular ICA and vertebral artery [VA]) to that of the MCA. The study addressed that the TCD of the cervical submandibular ICA and VA is a valid screening method to detect RLS due to a PFO^32^. However, most studies have replaced the temporal window due to a PTW with a suboccipital window to detect RLS using the TCD. The studies had demonstrated the usefulness of the suboccipital window (for monitoring the VA) in detecting RLS using the TCD^30,32^. Although these studies had determined that the suboccipital window had a diagnostic ability that was equal to that of the MCA, there are no quantitative studies detecting the extent of RLS, or comparing the temporal and suboccipital windows using correlation coefficients and reliable statistics.

In addition, the suboccipital window was similar to the temporal window in evaluating the PFO grade in a wide range (min 0 to max 509 MESs) as well as identifying the measured MESs at the same time^34,35^. Since the blood flow amount of the BA is quite equal to that of the MCA, the diagnostic ability for detecting RLS may be similar in the TCD monitoring. The amount of blood in the vertebrobasilar system is lower than that (about 20%) in the carotid circulation^36^. A study had evaluated the flow in the proximal and distal cerebral arteries using high-resolution phase-contrast magnetic resonance imaging. The study showed that the total cerebral blood flow (717 ± 123 mL/min) was distributed (on each side) as follows: middle cerebral artery, 21%; distal MCA, 6%; anterior cerebral artery (ACA), 12%; distal ACA, 4%; ophthalmic artery, 2%; posterior cerebral artery, 8%; and basilar artery, 20%^37^, which may be consistent with our investigations of a similar blood flow amount between MCA and BA. Therefore, the suboccipital window might be a better alternative than the submandibular ICA or orbital window in patients with PTWs. Several studies have shown that ischemic lesions are more common in the posterior cerebral circulation of patients with RLS found using the Valsalva maneuver^29^. However, we did not show that MESs was more common in the posterior cerebral circulation of patients during the Valsalva maneuver.

The suboccipital window using BA is as convenient as the temporal window when diagnosing RLS in TCD monitoring. A quantitative study conducted by Guo et al. that compared the left MCA and the left VA for the detection of RLS showed no significant differences between the two (including constant and provoked RLS)^30^. However, Guo’s study had also several limitations. First, the probe for VA monitoring was manually positioned. The VA might have been missed while testing if its course had changed. Second, The VA has many variations including hypoplasia^38,39^. VA hypoplasia (VAH) is not an uncommon congenital variation of the VAs leading to asymmetry. VAH with caliber discrepancies of more than 1:1.7 was observed in up to 10% of normal individuals^38,39^. As it is difficult to monitor the VA using the TCD under the same condition, it can be difficult to detect RLS. As in this study using BA, it is easy to fix the probe through the headset and there is no need to consider VA hypoplasia. It was much easier to use as the sonographer did not have to fix the probe manually during TCD study in the suboccipital window. In this study, MESs were measured only on the supine position, our method allows us to investigate further patients with prone and sitting postures. Some studies have shown that the upright sitting position is the best position for detecting RLS^40,41^. This was relatively more comfortable and easily achieved. Therefore, we chose the BA instead of VA and compared it to the MCA in our study.

Previous randomized controlled trials did not reveal any statistical significance between PFO closure and medical therapy for reducing stroke rates^42–44^. However, some recent studies have suggested that PFO closure is more effective than medical therapy for reducing recurrent stroke rates^4–7^. The benefits observed in recent trials following PFO closure is probably related to several factors involved in the selection of patients with PFO features, such as a large shunt size, those with a more severe presentation or presence of an atrial septal aneurysm^4,6,7^. Therefore, it is important to evaluate the size and the severity of the PFO with RLS. TCDs could be an alternative to TEE for assessing the severity of PFO and a useful follow-up test for patients after PFO closure. We believed that TCD monitoring for BA can also be a method for quantitatively assessing the severity of PFO.

Our study has several limitations. First, the MCA and BA were compared only in provoked RLS. A comparative study of the MCA and BA is needed in constant RLS. Second, this study has not proven that ischemic lesions are located predominantly in the vertebrobasilar circulation, especially in patients with provoked RLS^29^. A previous study showed that the time taken for appearance of the bubble after administering the injection was higher in the vertebrobasilar circulation^31^. In future studies, the number of bubbles in addition to the time of occurrence of the bubbles needs to be considered while comparing the MCA and BA. Third, the BA may also present with hypoplasia and have lower blood flow than that in the carotid circulation. This may be associated with bilateral VAH or bilateral fetal-type posterior circle of Willis. We identified 21 patients with BA hypoplasia (diameter < 2 mm) in our study. Even with the basilar artery hypoplasia, the weighted kappa index was 0.896 when categorized using the SLS in Tier 3. Additionally, there were no patients in whom the BA was not detected using the TCD.

In conclusion, the suboccipital window using BA in RLS diagnosis is as valuable as the temporal window and sonographers can easily perform TCD monitoring in detecting RLS in patients with PTW.

## Methods

### Participants

This retrospective study was reviewed and approved by the Institutional Review Board (IRB) of Ajou University Hospital, Suwon, Korea (AJIRB-MED-MDB-21-349). The Declaration of Helsinki, was followed. The requirement for informed consent was waived by IRB of Ajou University Hospital owing to the retrospective nature of this study. The stepwise TCD method was our routine protocol for detecting RLS in our hospital.

**Figure 2:**
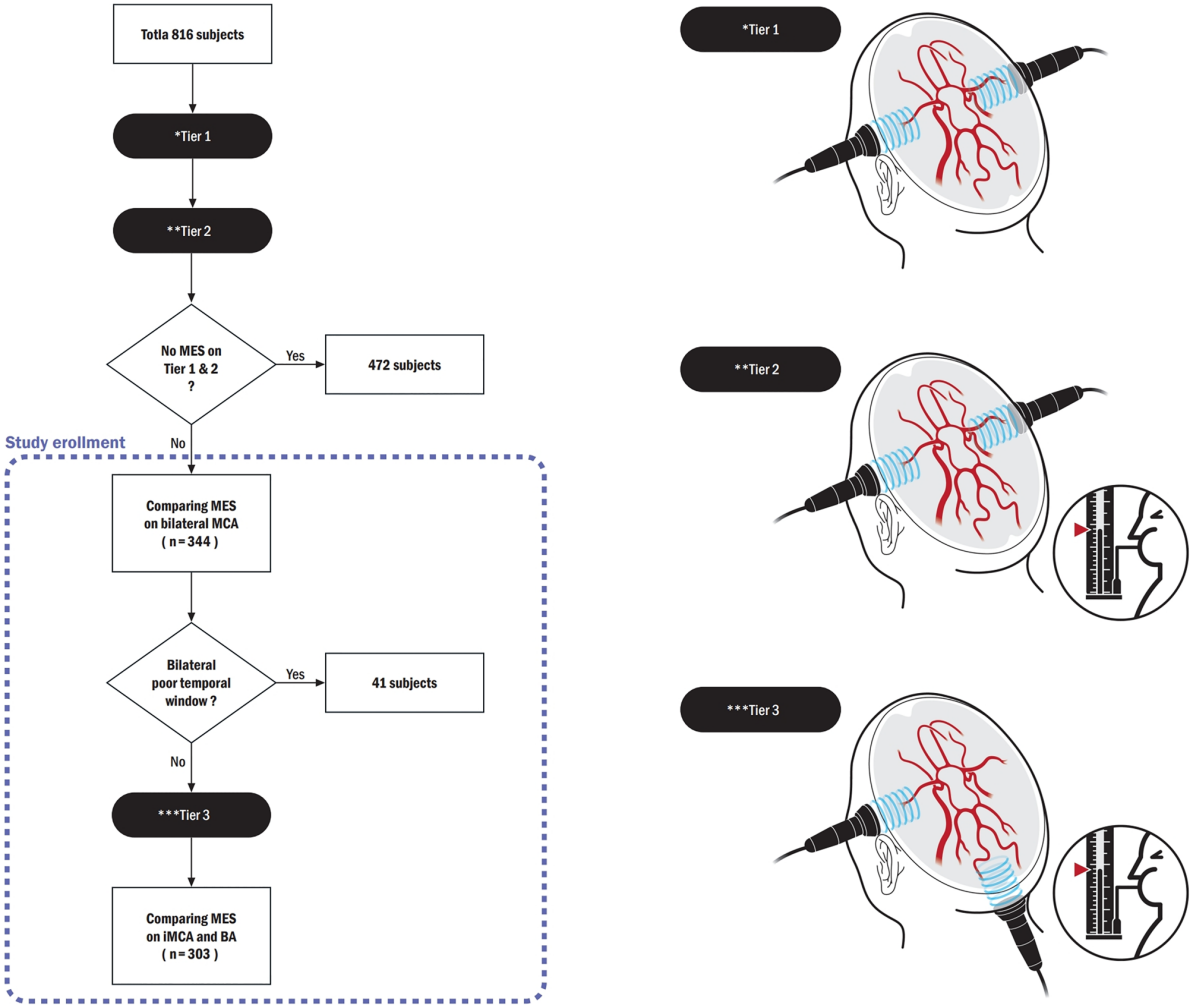
Flow diagram of study enrollment. Tier 1*: Bilateral MCA (or ICA) monitoring on rest state. Tier 2**: Bilateral MCA (or ICA) monitoring with Valsalva maneuver. Tier 3***: Simultaneous monitoring of iMCA and BA with Valsalva maneuver. *MES* microembolic signals, *PTW* poor temporal window, *MCA* middle cerebral artery, *BA* basilar artery, *iMCA* index of MCA, *ICA* internal carotid artery.

Patients tested using agitated saline TCD from December, 2015 to September, 2020 were recruited in this retrospective study. A total of 816 patients with headaches, TGA, WMHI, TIA, or stroke were tested using the TCD at Ajou University Hospital. The study excluded patients who did not reveal any MESs on TCD while in the resting state or on performing the Valsalva maneuver (n = 472). Therefore, a total of 344 patients were included in the study. Figure 2 shows the stepwise TCD method for the recruitment of this study population.

### The TCD procedure

The tests were performed in the following manner. An evaluation tool with three tiers was instituted to evaluate MESs during TCD monitoring. In tier 1, the TCD test was performed in both the right and left MCAs (or internal carotid arteries [ICAs] with a PTW) in the resting state. In tier 2, the TCD test was carried out after administering agitated saline for > 3 min while performing the Valsalva maneuver. In tier 3, if one or more MESs were detected from tier 1 or 2, MCA on one side (termed as the index MCA [iMCA]) was tested along with BA for > 3 min after performing the Valsalva maneuver. However, patients with bilateral PTWs were excluded 41 in tier 3 (Fig. 2).

Patients were advised to limit coffee and tobacco during 4 h before the examination, and also recommended to stop anticholinergic drugs during 48 h in advance to the TCD examination. In addition, to reduce the effect of the drug, patients who scheduled an examination were tested in the morning. The patients were asked to exhale into a manometer and maintain a pressure of 40 mmHg for 10 s to trigger the Valsalva maneuver. The iMCA was defined as MCA on one side, either right or left that had better visibility or detectability for MES. Microbubbles were provoked by administering 9 mL of 0.9% saline mixed with 1 mL of the patient’s blood and 1 mL of air. All 344 patients underwent TCD examinations as described in tiers 1 and 2. TCD as defined in tier 3, was performed in a total of 303 patients after excluding those with bilateral PTWs (n = 41).

**Figure 3:**
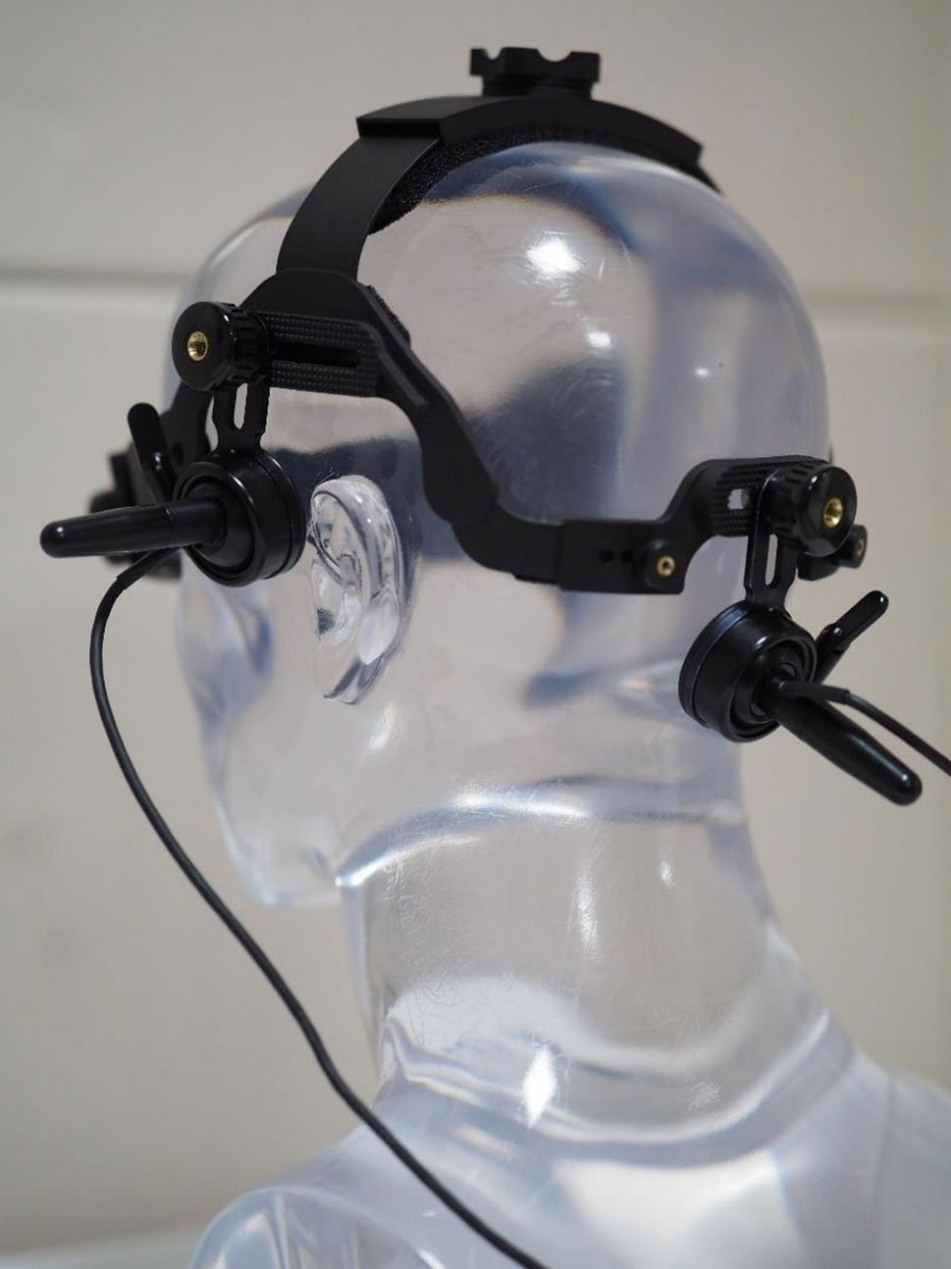
Fixable device which can be placed on the MCA and BA during TCD of the PFO.

A TCD examination was performed by sonographers using the PMD 150 M (Spencer Technologies, Seattle, WA) or TC8080 (Pioneer TC 8080; Viasys Healthcare, Madison, WI) with a 2 MHz probe. MES were recorded using probes mounted on the modified headset (Phifix; Astron, Suwon, Republic of Korea) that was monitored for both MCA and BA at the same time in detecting RLS using TCD (Fig. 3). There are two probes on the headset. One probe is placed on the temporal window to detect the MCA and the other probe is placed on the suboccipital window to detect the BA (Fig. 3). When the iMCA and BA were monitored simultaneously for MES using TCD, the patients were instructed to wear the headset and perform the Valsalva maneuver. During examination, the patient was placed in a supine position, and when measuring MES as BA and MCA using TCD, the patient turned his head to a comfortable direction and the test was performed. MES was defined as visible (short duration, high-intensity and unidirectional) and audible (click, chirp, or whistles) signals in the Doppler flow spectrum. All the MES were reviewed by two neurologists and sonographers of more than 10 years’ experience.

### MES grading

We used two grading systems for categorizing the MESs: the International Consensus Criteria and Spencer’s Logarithmic Scale (SLS)^34,35^. The patients were divided into four groups based on the MES using the International Consensus Criteria as follows: Grade 0, no occurrence of microbubbles; Grade 1, 1–10 microbubbles; Grade 2, > 10 microbubbles and shower pattern; Grade 3, curtain pattern^34^. The MESs were also categorized into six groups based on the SLS: Grade 0, no occurrence of microbubbles; Grade I, 1–10 microbubbles; Grade II, 11–30 microbubbles; Grade III, 31–100 microbubbles; Grade IV, 101–300 microbubbles; Grade V, > 300 microbubbles^35^.

### Statistical analysis

Statistical analyses were performed using the R Statistical Software (version 3.6.3.; R Foundation for Statistical Computing, Vienna, Austria). To verify the consistency of the agreement tests in various tiers, the analysis was performed in two ways. First, the weighted kappa index was used to compare the agreement between the right and left MCA, and the agreement between the iMCA and BA after classification based on the International Consensus Criteria and SLS in tiers 2 and 3. Second, MES identified quantitatively in the left and right MCA were verified and compared using the intraclass correlation coefficient (ICC) in tier 2. Similarly, the microbubbles identified in the iMCA and BA were verified, and compared using the ICC in tier 3. P < 0.05 was considered statistically significant.
